# Public Understanding of Pandemic Influenza, United Kingdom

**DOI:** 10.3201/eid1210.060208

**Published:** 2006-10

**Authors:** Ravindra K. Gupta, Martina Toby, Gagori Bandopadhyay, Mary Cooke, David Gelb, Jonathan S. Nguyen-Van-Tam

**Affiliations:** * Centre for Infections, London, United Kingdom;; † University of Sheffield Medical School, Sheffield, United Kingdom;; ‡ University of Nottingham Medical School, Nottingham, United Kingdom

**Keywords:** Communicable diseases, influenza, virus, pandemic, risk communication, public awareness, letter

**To the Editor:** Widespread outbreaks of influenza A (H5N1) in poultry and severe infections in humans have raised the possibility of an influenza pandemic. The 3 influenza A pandemics of the 20th century (*1*) were associated with considerable socioeconomic disruption as well as many deaths and pressure on health services. Experiences in the United States during the 1918–1920 pandemic (*2*) suggest that government advice that conflicts with personal or societal beliefs may not be followed, thus jeopardizing public health measures. Experience from the outbreak of severe acute respiratory syndrome has highlighted some pitfalls in achieving public understanding (*3*) and compliance (*4*) in the era of mass communication. Even if initial compliance is achieved, previous behavior patterns may reemerge during a pandemic as people begin to perceive that they have little control over the threat (*5*) or reduce their estimation of the risk (*6*).

Building robust public understanding has been made a priority in preparedness and response plans (*7*). However, despite widespread media coverage, little attention has been paid to assessment of public knowledge about the threat for pandemic influenza and surrounding issues. Such information may be essential to optimize public education strategies.

A questionnaire-based population survey was administered in March 2005 by 2 of the authors (MT and GB) to identify public knowledge about pandemic influenza, awareness of its potential effects, key information needs, and willingness to follow advice about public health measures. A structured interview consisting of 20 questions was used. Participants were approached at random and interviewed (in English) in public places including parks, shopping malls, libraries, and train stations in northern London. This area has considerable ethnic diversity (55% of the population is nonwhite) and a socioeconomic status similar to the rest of London. Recruited participants were >18 years of age and resided in the United Kingdom. They were excluded if another family member had previously completed the survey. Age and sex ratios were selected to reflect population centiles calculated from the 2001 UK population census. Statistical analyses were conducted with Fisher exact tests and epidemiologic tabulations in Stata version 8.2 (Stata Corp., College Station, TX, USA).

Of 273 persons approached for interview, 225 accepted and were eligible. Nine questionnaires were incomplete and therefore excluded, leaving 216 (79%) for analysis. Demographic characteristics of participants are summarized in the Table A1. Half the respondents chose the correct definition of a pandemic from 5 options. Statistical analysis demonstrated that those 32–44 years of age were more likely than those of other age groups to choose correctly (p = 0.001). Persons who left school at ages >17 years were more likely than those who left school earlier to select the correct answer (p = 0.007).

Sex of the respondent did not influence correct response; 56% of those 18–31 years of age versus 86% of those >60 years of age were aware of the threat of pandemic influenza (p = 0.006). When asked the likelihood of a pandemic during the next 10 years, 71% responded that it was likely or very likely, whereas 16% considered it unlikely or very unlikely. When offered a list of 4 possible negative affects identified by experts (healthcare service, food distribution, fuel distribution, and disruption to tourism), only one fourth thought that all 4 would occur. Details about symptoms of pandemic influenza were most frequently cited as the main public information need in the event of a pandemic. Television was rated by 68% of respondents as their preferred means of receiving information during a pandemic. Almost all respondents (97%) would wash their hands >5 times each day if requested, and 86% would definitely or probably be willing to stay away from public gatherings (unspecified) if asked. However, only 61% would stay away from work (unspecified period) as a means of avoiding pandemic influenza.

As far as we know, this is the first population-based study of knowledge and understanding of pandemic influenza. Public understanding of this threat and its potential effect in the United Kingdom appears to be limited. Our findings that older adults are more aware than younger persons has also been found in other settings (*8*) as has the increased public health awareness in more educated groups (*9**,**10*). Economic considerations retain high importance even with a potentially fatal threat, a phenomenon that has been previously noted with regard to self-quarantine (*4*). Our study did not address whether reluctance to take time off from work was more likely to be associated with public or private sector employment or self-employment. Further study in this area would help preparedness strategy.

This study was limited by a relatively small sample size, and its setting in 1 region of London may have implications regarding the extent to which the findings are applicable elsewhere. Further, larger assessments are needed both before and after specific pandemic influenza awareness programs as part of the ongoing process of pandemic preparedness.

## Appendix

**Table A1:** Demographic characteristics of study participants

Characteristic		No. male (%)	No. female (%)	Total (%)*
Total		93 (43)	123 (57)	216 (100)
Age, y				
	18–31	32 (34)	41 (33)	73 (34)
	32–44	25 (27)	32 (26)	57 (26)
	45–60	16 (17)	26 (21)	42 (19)
	>60	20 (22)	24 (20)	44 (20)
Age when left school, y				
	<16	30 (32)	33 (27)	33 (29)
	17–18	21 (23)	40 (33)	61 (28)
	>19	35 (38)	47 (38)	82 (38)
	Still in school	7 (8)	3 (2)	10 (5)
Occupation				
	Employed	58 (62)	79 (64)	137 (63)
	Unemployed	0	1 (1)	1 (0.4)
	Student	10 (11)	13 (11)	23 (11)
	Homemaker	1 (1)	9 (7)	10 (5)
	Retired	24 (26)	21 (17)	45 (21)

*Some totals may not equal 100% because of effects of rounding.
